# The Newly Identified T Helper 22 Cells Lodge in Leukemia

**Published:** 2015-07-01

**Authors:** Gholamreza Azizi, Mohsen Rastegar Pouyani, Shadi sadat Navabi, Reza Yazdani, Fatemeh Kiaee, Abbas Mirshafiey

**Affiliations:** 1 Imam Hassan Mojtaba Hospital, Alborz University of Medical Sciences, Karaj, Iran; 2 Research Center for Immunodeficiencies, Children’s Medical Center, Tehran University of Medical Sciences, Tehran, Iran; 3 Department of Immunology, School of Public Health, Tehran University of Medical Sciences, Tehran, Iran; 4 Department of Immunology, Faculty of Medicine, Isfahan University of Medical Sciences, Isfahan, Iran

**Keywords:** Th22, IL-22, Leukemia, AML, ALL

## Abstract

Leukemia is a hematological tumor in which the malignant myeloid or lymphoid subsets play a pivotal role. Newly identified T helper cell 22 (Th22) is a subset of CD4^+^ T cells with distinguished gene expression, function and specific properties apart from other known CD4^+^ T cell subsets.Th22 cells are characterized by production of a distinct profile of effector cytokines, including interleukin (IL)-22, IL-13, and tumor necrosis factor-α (TNF-α). The levels of Th22 and cytokine IL-22 are increased and positively related to inflammatory and autoimmune disorders. Recently, several studies have reported the changes in frequency and function of Th22 in acute leukemic disorders as AML and ALL. This review discusses the role of Th22 and its cytokine IL-22 in the immunopathogenesis of leukemic disease.

## Introduction

 Leukemia is a type of cancer that affects the bone marrow and/or blood cells. In patients with leukemia, there is disturbance in the normal development of the blood cells and abnormal, immature blood cells crowd out normal blood cells. In such patient, the full blood picture shows abnormal blood counts. These patients have anemia and exhibit low red blood cells, hemoglobin, and platelet counts. The leukocyte count may be low or high but the patients usually have neutropenia. As leukemia is a disseminated disease it also produces a wide variety of other symptoms like: bone pain, lymphadenopathy, hepatosplenomegaly, rheumatoid arthritis fever, nephritis, meningitis and hyperparathyroidism^.^^1^ The type of leukemia is characterized by where the cell is in the stage of development when it becomes malignant, or leukemic. Leukemia can be started in both myeloid and lymphoid cells. Commonly, there are four main types of leukemia: Acute myelogenous leukemia (AML), Chronic myelogenous leukemia (CML), Acute lymphocytic leukemia (ALL), and Chronic lymphocytic leukemia (CLL). ^2^^-^^4^  AML and ALL progress much faster and symptoms may worsen more rapidly than the chronic leukemia (CML and CLL). It should be noted that diseases of the lymphoid or myeloid immune cells change the normal functioning of the host immune system, rendering it unable to mount an immune response.^   5 ^ It is demonstrated that similar to other cancers, development of leukemia is associated with immune suppression in the host, contributing to the defect to mount an effective immune response against the leukemic cells. Some data suggest the immune suppression may be a serious factor in increasing AML risk in experimental settings, whereas immune stimulation may be useful in treating AML^        6 ^ Gale et al. suggest immune suppression increases risk of developing AML and that this risk is even higher following intense extended immune suppression.^   7^

Although there has been a quick increase in our knowledge about the malignant cells' biology in leukemia, investigators still lack a clear understanding of the role of non-malignant lymphocytes specially CD4^+^ T cells in the pathogenesis of leukemia.^        8 ^ Rare evidence shows that in AML patients, T helper (Th) type 1 cells are significantly decreased in newly-diagnosed or relapsed/refractory patients compared with complete remission or healthy persons, which is consistent with the reduced immune function.^        9 ^ In addition, it was known that interferon-gamma (IFN-γ), as the main cytokine of Th1 subset, sensitizes human myeloid leukemia cells to apoptosis by a pleiotropic mechanism.^        10 ^ However, in contradiction to these, Zaki et al. demonstrated not only IFN-γ has anti-apoptotic effect on the neoplastic B cells but also there are increased numbers of IFN-γ-expressing CD4 and CD8 T cells in CLL patients.^   11 ^ On the other hand, data indicate that interleukin (IL)-17, the main effector cytokine of Th17 cells in inflammatory and autoimmune diseases, has tumor-promoting effects, especially in the context of inflammation. ^12^^,^^13^  At present, existing data about correlation of Th17 cells and IL-17 with acute leukemia are controversial. Some data have revealed the elevated Th17 levels in newly-diagnosed AML patients whereas others have shown normal levels in newly-diagnosed AML patients.^14^^,^^15^ Th22, as the newest identified subset of Th cells is clearly separated from Th17 and Th1 subsets with a different identity with respect to function and gene expression.^        16 ^ Novel findings have shown that Th22 and its effector cytokine IL-22 are also implicated in the pathogenesis of inflammatory and autoimmune diseases, myelodysplastic syndrome (MDS) and leukemias as AML and ALL. ^17^^-^^21^ In this review, we discuss the frequency and role of Th22 cell and its important cytokine, IL-22, in leukemia.

**CD4 T cell subsets**

The lymphocyte linages in human are mainly comprise of thymus-derived T lymphocytes and bone-marrow-derived B lymphocytes, as well as a non-T, non-B lymphocyte known as Natural-Killer cell (NK cell) which is a type of cytotoxic lymphocyte critical to the innate immune system.^   22 ^ CD4^+^T cells along with CD8^+^T cells make up the majority of T lymphocytes.^        23 ^ In 1986, Mosmann et al. identified 2 subtypes of activated CD4^+^ T cells, Th1 and Th2 cells, which differed from each other in function and type of cytokine profile. ^24^^,^^25^  In addition to classical Th1 and Th2; other subsets have been identified including Th3 (a regulatory subset), Th9, Th17 and Th22. Each phenotype is characterized by a specific cytokine profile, unique signaling pathways and expression of lineage-specific transcription factors (notably T-bet for Th1, GATA-3 for Th2, Retinoid-related orphan receptor (ROR) alpha and RORγt for Th17 and the aryl hydrocarbon receptor (AHR) for Th22 cell) as well as epigenetic modifications at appropriate genes. The effector functions of these cells are mediated by the cytokines secreted from the differentiated cells.^        26^

Th1 cells are a subset of CD4^+^ T cells and a part of the adaptive immune system. Th1 cells, which produce IFN-γ, IL-2 and tumor necrosis factor-α (TNF-α), arouse cell mediated immunity and are the main effective cells against different types of intracellular pathogens. ^27^^,^^28^  In addition, they tend to produce pro-inflammatory cytokines and are involved in the formation of organ-specific autoimmune disease. Th1 polarization is induced by the cytokines IL-12 (most important), IL-15, IL-18 and IFN-α, which are present in the inflammatory milieu. Feili-Hariri et al. suggest that IL-12 alone, although is necessary for Th1 differentiation, but is not sufficient to induce Th1 responses and needs other cytokines as granulocyte macrophage-colony stimulating factor (GM-CSF) and IL-15. These cytokines are normally produced by the dendritic cells (DCs) or the NK cells. ^29^^,^^30^ 

Th2 cells are thought to have roles as protection against parasitic helminths. Th2 cells are also involved in the pathogenesis of allergic rhinitis, food allergy, and other allergic diseases. ^27^^-^^31^  In addition, Th2 cells which produce IL-4, IL-5, IL-6, IL-9, IL-10, and IL-13, induce strong antibody responses (immunoglobulin class switching in B cells) and eosinophil accumulation, whereas inhibit several functions of phagocytic cells. Th2 response is induced by production of IL-4, which in turn is secreted by the naive T cells or the mast cells and/or macrophages.^32^^,^^33^ T-bet and GATA-3 are two transcription factors responsible for the shift of naive CD4+ T cells into the Th1 or Th2 phenotype, respectively.^        34^

**Th3 cells**

 are one of Treg subtypes that can diﬀerentiate from naive CD4+T cells following ingestion of a foreign antigen through the oral route.^   35 ^ For the first time Th3 cells were identiﬁed in mice following oral administration of myelin basic protein (MBP) for tolerance induction.^        36 ^ It is demonstrated that treatment with MBP leads to induction of TGF-β-producing Th3 cells which are MBP speciﬁc and inhibit the progression of multiple sclerosis mouse model.^        37 ^ Therefore, regulatory Th3 cells are a unique T cell subset induced by orally administered antigens in vivo and triggered in an antigen-specific manner. They mainly secrete TGF-β, provide help for IgA production, and have suppressive effects on both Th1 and Th2 cells. ^38^^,^^39^  There are controversial findings about FoxP3 expression in Th3 cells. Although some studies have detected FoxP3 in Th3 cells; others indicate that Th3 cells do not express this transcription factor.^40^

**Th9 cells**

The most recently defined subset of T-helper cells, are important for host defense against extracellular pathogens as parasitic helminths. However, they may also have a role in multiple diseases including chronic allergic inflammation, asthma and airway remodeling, and autoimmune skin diseases as psoriasis.^41^^,^^42^ When Th0 cells are exposed to TGF-β and IL-4 they differentiate into Th9 cells which produce IL-9. IL-9 is the characteristic cytokine of Th9 cells. TGF-β and IL-4 induce the expression of the transcription factors PU.1/Spi-1 and Interferon Regulatory Factor 4 (IRF4), which subsequently regulate expression of the IL-9 gene. Signaling mechanisms and transcription factors involved in Th9 differentiation are still being investigated. It is known that Th9 cells are increasingly linked to Th2 cells, and there is a significant plasticity between Th9 and Th2 cell types. IFN-γ suppresses the activation of Th9 similarly to Th2 cells but unlike Th2 cells, Th9 cells do not express IL-4, IL-5, or IL-13.^43^^-^^46^

**Th17 cells**

 are a lineage of CD4^+^ effector T cells which is distinct from the Th1 and Th2 lineages.Th17 cells have evolved primarily to defend against bacterial, mycobacterial and fungal pathogens.^   47 ^ They are also believed to be involved in autoimmune disorders such as rheumatoid arthritis (RA),^48^ systemic lupus erythematosus (SLE), ^        49 ^ Sjogren's syndrome,^        50 ^ psoriasis,^        51 ^ ankylosing spondylitis,^        52 ^ and Behcet's disease.^        53 ^ Th17 cells produce IL-17, IL-17F, IL-21 and IL-22, which induce a massive tissue reaction owing to the broad distribution of the IL-17 and IL-22 receptors. IL-17 is thought to be important for the neutrophil recruitment as it up-regulates CXCL8 expression. The differentiation factors for Th17 include TGF-β plus IL-6 or IL-21. Th17-specific transcription factors are signal transducer and activator of transcription 3 (STAT3), RORγt and RORα which are involved in the development of Th17 cells. Moreover, it is demonstrated that IL-21 along with IL-23 signals are necessary for expansion and stabilization of Th17 phenotype. ^54^^-^^56^ 

**Th22 cells**

Th22 cells are a novel subtype of CD4^+^ T cells characterized by the secretion of both IL-22 and TNF-α as well as expression of C-C chemokine Receptor type4 (CCR4), CCR6 and CCR10. They are also able to express other mediators such as fibroblast growth factor (FGF) isoforms which are involved in tissue remodeling. Recently, it is suggested that IL-22 and TNF-α produced by Th22 cells, synergistically induce immunomodulatory mediators like: chemokine (C-X-C motif) ligand 9 or CXCL-9, CXCL-10, and CXCL-11, which are antimicrobial chemokines, as well as the antimicrobial peptide mBD-2 also known as murine β-defensin 2.^   57 ^ Therefore, these cells have little or no direct impact on other cells of the immune system, but exert selective effects on the epithelium. Although it is not clear if Th22 cells have a role in reproductive tract mucosal immunity, it has been illustrated that cytokines of Th22 play an important protective role in reproductive tract infection. Th22 cell migration into the skin is driven by surface expression of CCR4 and CCR10. ^57^^,^^58^  Th22 lymphocytes do not express IFN-γ (Th1 marker), IL-4 (Th2 marker) as well as IL-17, IL-23 receptor, CCL20, CD161 (Th17 markers). Moreover, Th22 cells are distinct from Th17 cells due to a high degree of poly functionality and low expression of CD161.^        59 ^ These characteristics concertedly, distinguish Th22 cell as a new lineage of helper T cells which is distinct from the other Th subtypes. ^16^^,^^60^  In the presence of two cytokines: TNF-α and IL-6, newly activated naive CD4^+^ T lymphocytes can differentiate into Th22 cells, but this differentiation could be inhibited by increasing doses of TGF-beta.^   20 ^ Also Th22 cells do not express T-bet, GATA-3 and RORγt which are specific transcription factors of Th1, Th2 and Th17, respectively.^61^ However, Duhen et al. (2009) reported that there is low or undetectable expression of these transcription factors in Th22 cells.^        61 ^ It seems that Th22 expansion is regulated by a discrete transcription factor called the aryl hydrocarbon receptor (AHR), though intracellular molecular mechanisms implicated in Th22 differentiation remain to be fully characterized and additional intracellular molecules are still being investigated.^        62 ^ Alam et al. (2010) showed that in CD4^+^ T lymphocytes Notch signaling drives IL-22 secretion by stimulating the AHR. Furthermore, even in the absence of STAT3, Notch-mediated stimulation of CD4^+^ T cells increased IL-22 production.^        63 ^ Baba et al. illustrated that VAF347, an AHR ligand, promotes Th22 development by selectively acting on monocytes and naive CD4^+^Th cells. Also it suppresses the generation of the either Th1 or Th17.^        62 ^ In an analogous research, it was demonstrated that dioxin, a high affinity and stable AHR-ligand, as well as the natural AHR-ligand 6-formylinolo (3, 2-b) carbazole induced P450A1 which is the down-stream target cytochrome of AHR, and without affecting IFN-γ, they augmented IL-22 production whereas simultaneously reduced IL-17A production by CD4^+^ T cells. In addition, the specific AHR-inhibitor CH-223 191 suppressed these effects. Importantly, following AHR-ligation not only the number of Th17 cells decreased but also naive CD4^+^ T cells were primed to produce IL-22 without IL-17 and IFN-γ.^        64 ^ This affirms previous results of Trifari et al. who showed that RNA interference (RNAi)-mediated downregulation of either AHR or RORC would affect IL-22 production, while IL-17 production was affected just by RNAi-mediated down-regulation of RORC. Besides, AHR agonists considerably changed the IL-22- versus IL-17-producing cell balance.^        58^

Naive CD4^+^ T cells could differentiate into Th22 cells in the presence of both types of DCs namely conventional DCs (cDCs) and plasmacytoid DCs (pDCs). Accordingly, it has been revealed that for the expansion of Th22 cells, pDCs are more powerful than cDCs.^        58 ^ Generally it has been shown that naive CD4^+^ T cells could differentiate into Th22 cells when co-cultured with pDCs in the presence of TNF-α and IL-6. However, its mechanism is still not clearly understood. Both of these cytokines are commonly secreted by DCs during maturation phase. In addition, Langerhans cells of the epidermis as specialized and professional antigen-presenting cells (APCs) are capable of inducing Th22 cells.^58^^,^^61^^,^^65^ Accordingly, in a recent study, Duluc et al. (2013) revealed that both CD14^-^ lamina propria DCs and Langerhans cells are potent inducers of Th22 lymphocytes in the human vaginal mucosa.^        66 ^ In vitro, Th22 cell clones seem to be fully stable and maintain expression of IL-22 but no other cytokines related to other CD4^+^Th cells when cultured in Treg-, Th1-, Th2-, and Th17-polarizing conditions.^        16 ^

**IL-22**

IL-22 is structurally an IL-10- related cytokine that is secreted by innate immune cells, Th17 cells and Th22 cells. It was first described as a cytokine associated with Th1 cells in mice and humans. ^58^^,^^67^  Latest data have demonstrated that IL-22 production is more closely related to IFN-γ than IL-17 among CD4^+^ T lymphocytes; indicating that during differentiation of CD4^+^ T lymphocytes IL-17 and IL-22 are differentially regulated.^   68 ^ IL-22 is also produced by CD8^+^ T cells and IL-22^+^CD8^+^ T cell frequency correlates with the severity of atopic dermatitis disease.^        69 ^ In addition, other innate immune cells including CD11c^+^ DCs, NKT cells, γδ T cells and NK22 cells are capable of producing IL-22.^        70 ^ In response to TNF-α and IL-6, Th22 cells particularly in the skin produce IL-22, while, as reported in murine models of psoriasis, γδ T cells (as innate immune cells) produce IL-22 in response to IL-23 not only in the lung but also in the skin.^        71 ^ NK cells produce IL-22 in response to IL-12 and IL-18 or IL-23.^        72 ^ Although IL-22 is produced by immune cells, the data have shown that IL-22 receptor is not expressed by neither resting nor activated immune cells, and under both in vivo and in vitro circumstances IL-22 has no effect on these cells Conversely, IL-22 regulates the function of some tissue cells. For example: skin, kidney, pancreas, small intestine, liver, colon, and respiratory system cells are putative targets of this cytokine.^   73 ^ Hence, IL-22 expression allows Th22 cells to act on non-hematopoietic cells where the Th22 cells seem to have a protective role in wound repair/ healing regulation of the skin, lung and gut.^        61 ^ Some cell types, particularly epithelial cells, express IL-22 receptor (IL-22R) whose stimulation upon recognition of IL-22 results in the production of molecules which are both locally and systemically active.^74^

In 2002, Lejeune et al. proposed that following the activation of signal transduction pathways in response to IL-22, some signaling molecules including Tyk2 and JAK1 but not JAK2 are activated and the transcription factors STAT1, STAT3, and STAT5 will be phosphorylated. In addition, by using specific monoclonal antibodies against the phosphorylated form of ERK1/2, MEK1/2, JNK, p90RSK, and p38 kinase, they showed that IL-22 activates the three main MAPK pathways.^        75 ^ While some T cell transcription factors including STAT3 and RORγt stimulate the expression of both IL-22 and IL-17 cytokines, other factors, like c-Maf specifically act on IL-22.^   76 ^ Altogether, IL-22 does not communicate between cells of the immune system but is a T cell secreted cytokine which directly enhances the nonspecific immunity of the aforementioned tissues.^   73^

Despite its positive effects for the host against many infections, IL-22, depending on the target tissue, can be pathogenic because of its intrinsic pro-inflammatory properties. It has been shown that deleterious effects of IL-22 could be further enhanced when this cytokine is secreted together with other inflammatory cytokines, specially IL-17.^   76 ^ Furthermore, IL-22 is known to be expressed by Th22 cells in many chronic autoimmune diseases, including Rheumatic disease,^77^^,^^78^ Psoriasis,^   79 ^ Multiple Sclerosis,^        80 ^ Guillain-Barré syndrome,^        81 ^ Immune Thrombocytopenia ^82^^,^^83^  and acute coronary syndrome,^   84 ^ as well as other disorders like aplastic anemia,^   85 ^ myelodysplastic syndrome,^        19 ^ gastric cancer,^        86 ^ acute lymphoblastic leukemia,^   18 ^ and acute myeloid leukemia.^   21 ^

**Th22, IL-22 in leukemia**

Th22 and also IL-22 play a role in tumor diseases. In 2012 Liu et al. suggested that circulating Th22 cells as well as Th17 cells are significantly increased in the peripheral blood of gastric cancer patients with tumor progression. It is also shown that the increased intratumoral IL-22-producing CD4^+^ T cells and Th22 cells correlate with gastric cancer progression and predict poor patient survival. ^86^^,^^87^  It is demonstrated that IL-22 expression and signaling is dysregulated in patients affected by many common cancers including skin, liver, gut and lung. In addition, apart from other cytokines associated with cancer, IL-22 has been restricted to tissue targets and its receptor (IL-22R1) is particularly expressed on epithelial and tissue cells, but not on benign immune cells.^   88 ^ However, it is demonstrated that expression of IL-22R1 and autocrine IL-22 stimulation contribute to lymphoid malignancy and tumorigenicity in anaplastic large cell lymphoma (ALCL).^        89 ^ The expression of IL-22R1 in anaplastic lymphoma kinase-positive (ALK^+^) ALCL is aberrant, whereas this receptor is absent in benign lymphocytes.^        90^

ALCL is a peripheral T-cell lymphoma that can present with either a T cell phenotype or null-cell phenotype. ALK^+^ALCL is characterized by the expression of IL-22R1 on the lymphoma cells and occurs mainly in pediatric and young adult patients.^90^^,^^91^ ALK^+^ALCL patients generally show clinical symptoms along with an inflammatory syndrome, including high fever, lymphadenopathy, and neutrophilia. In a study by Savan et al. it is reported that IL-22 plays a key role in inflammation as a result of aberrant expression of IL-22R1 on lymphocytes. Moreover, it is revealed that IL-22R aberrant expression may promote development of Th17 cells in ALK^+^ALCL. It is demonstrated that ALK^+^ALCL cell lines expressing IL-22R1, have a phenotype similar to Th17 cells which produce IL-22 and IL-17 at high levels in vitro. Also in ALK^+^ALCL patients the serum levels of IL-22 and IL-17 are increased.^        89 ^ In transgenic mice model, it is shown that autocrine amplification of IL-22, and the inflammatory phenomenon observed in these mice correlates with the up-regulation of serum granulocyte colony-stimulating factor (G-CSF) and IL-17. These data show that IL-22R1 expression on lymphocytes can amplify circulating IL-22 in vivo, identifying the role of this cytokine as a potent principal player in ALK^+^ALCL.^        89 ^

There is raising evidence indicating that some non-hematopoietic cancers may use the IL-22R1/STAT3 pathway to increase their growth and survival. A recent study by Zhang et al. showed that lung cancer cell lines expressing IL-22R1 are protected from chemotherapeutic drug-induced apoptosis by autocrine IL-22 via activation of STAT3 and its downstream anti-apoptotic proteins.^        92 ^ In addition, the oncogenic role of STAT3 in ALK^+^ALCL has been widely studied, and it is suggested that STAT3 activation inhibits apoptosis in these cells.^        93 ^

Immunological disorders have shown to be related to the pathogenesis of some leukemias, as IL-22 is increased and correlates with CD38 expression in patients with B-chronic lymphocytic leukemia.^        94 ^ In addition, Th22 cells as the most important producers of IL-22 have been identified as major inducers of tissue inflammation and are thought to be implicated in the pathogenesis of some leukemic disorders. Currently, more studies explored the role of Th22 cells in the pathogenesis of some leukemias such as AML and ALL.^20^^,^^95^

In AML, the raise in Th subsets including Th22, Th17, and Th1 cells plays a pivotal role. However, the role of Th subsets in the immune pathogenesis of AML remains unclear. Liu et al. indicated that Th22 cells level and IL-22 expression in peripheral blood were significantly reduced in the newly diagnosed patients and complete remission patients compared to healthy controls indicating that Th22 might play a protective effect in AML.^   21 ^ However, as IL-22 is involved in the regulation of cell cycle control, cell growth and proliferation, it is possible that IL-22 might play a role during tumor genesis.  ^96^^,^^97^  Yu et al. in 2014 demonstrated that the plasma concentration of IL-22 and percentage of Th22 and Th17 subsets but not Th1 subset were significantly increased in AML patients. Moreover, RORC expression was significantly increased in AML patients with complete remission and non-complete remission. However, they reported a strange data about the plasma IL-17 level in AML patients, explaining that plasma IL-17 level was significantly decreased in newly-diagnosed AML patients with non-complete remission or complete remission compared with healthy subjects.^        95 ^ In another study Han et al. demonstrated that the frequency of Th17 cells was significantly increased in bone marrow mononuclear cells and PBMCs from AML patients compared with healthy donors. Furthermore, Th22 and IL-22 showed positive correlation with pure Th17 cells, but Th22 showed negative correlation with Th1 in newly-diagnosed AML patients. 

In newly-diagnosed AML patients, peripheral blood blast cell indicated positive correlation with Th22 and negative correlation with Th1 cells. In a newest study Sun et al. showed that many cytokines were abnormal in AML bone marrow microenvironment. In their study the significant differences on cytokine levels tested were observed among the newly-diagnosed AML patients, AML patients in complete remission and controls except IL-21 and IL-35 cytokines. In AML newly-diagnosed group, IFN-γ concentration positively correlated with IL-21 and IL-22 level.^   98 ^ Conversely, Han et al. reported that plasma levels of the cytokines IL-1β, IL-6, IL-17, IL-22, IL-23 and TGF-β1 were significantly increased in blood and bone marrow in AML patients compared with healthy control. Moreover, IL-1β, IL-6, and IL-23, but not TGF-β1 promoted the generation and differentiation of Th17 cells from naive CD4^+^ T cells in vitro.

 IL-17A induced the proliferation of IL-17R-positive AML cells via IL-17R, in which the activation of PI3K/ Akt and Jak/Stat3 signaling pathways might play principal roles. It should be noted that, combination of IL-17A and IL-22 significantly decreased the generation of Th1 cells and the production of IFN-γ from PBMCs of healthy controls or AML patients. AML patients with high Th1 cell frequency had prolonged survival, whereas patients with high Th17 cell frequency had poor prognosis.^   99 ^ In conclusion, these data represent that Th22, Th17 and their cytokines contribute to the pathogenesis of AML and might be a favorable and novel clinical index for AML.

Myelodysplastic syndrome (MDS) is a highly heterogeneous clonal hematological malignancy, having a high rate of progression to AML. Immunological mechanisms are increasingly recognized in the progression of MDS. Early-stage MDS is characterized by autoimmune-mediated myelosuppression whereas late-stage MDS involves immune evasion, giving dysplastic cells growth potential to progress into AML. Shao et al. demonstrated that in early-stage MDS, peripheral Th17 subsets were significantly elevated and correlated with peripheral Th22 subsets compared with healthy donor and late-stage MDS. In addition, significantly higher levels of peripheral Th22 expansion, mRNA expression of the cytokines IL-6, and TNF-α and also lower level of RORC mRNA expression were noticed in late-stage MDS compared with early-stage MDS. These data indicate that Th22 cells are involved in the dynamic immune responses of MDS. ^19^^,^^100^^,^^101^ 

In recent years the aim of studies has been to assess the function of T cells in patients with ALL. These studies confirm the involvement of cellular immunity in the ALL leukemic process. It is demonstrated that the profile of Th subsets is different in ALL patients and shows some correlations with clinical index, indicating that Th subsets may be implicated in the pathogenesis of ALL. Luczyński et al. in 2005 reported that patients at the time of ALL diagnosis exhibited higher percentages of T cells with adhesion molecule ICAM-1, an increased population of Th2 cells, and activation molecule CD38 expression compared with the healthy controls. During and after remission they showed elevated percentages of Th cells with IL-2R expression, a rise in Th1 cells producing IFN-γ, and a decreased population of CD38^+^ T cells. During fever and infection, higher levels of activated T cells, a rise in Th1, and no change in Th2 populations were observed. However, they did not study the function and frequency of Th22 cells and IL-22.^        102 ^ In 2012 Liu et al. investigated the frequency of Th22 cells in peripheral blood of patients with ALL and evaluated its significance. They found that the frequency and function of Th22 cells were reduced in ALL patients, and that Th22 cells might be negatively correlated with ALL progression. In ALL patients the lower levels of TNF-α and IL-6, and overexpression of TGF-β might suppress the differentiation of Th22 cells.^        103 ^ In a similar study in 2013, Cheng showed that the frequency of Th22 cells and the expression levels of IL-22, TNF-α and IL-22 mRNA in newly diagnosed patients and in patients with complete remission were significantly lower than healthy donors. Also the expression level of TGF-β in both aforementioned groups of patients was obviously higher than healthy donors. Meanwhile, Cheng reported that the expression level of IL-22 in newly diagnosed patients was positively related with expression level of TNF-α, and negatively related with TGF-β. It is concluded that decreasing Th22 cells and down-regulation of IL-22 expression maybe related with pathogenesis of ALL. It is also suggested that a low level of Th22 cells could be a risk factor for ALL.^20^^,^^103^ However, in controversy with the above-mentioned data, Tian et al. in 2014 suggested that Th22 and Th17 frequencies, serum IL-22 level and AHR expression were significantly increased in newly diagnosed or ALL patients with complete remission compared with healthy donors. In addition, Th22 showed a positive correlation with Th17 or Th1 cells in ALL patients. Moreover, similar to AML patients a significant decrease of IL-17 level, Th1 frequency and T-bet expression was observed in newly diagnosed ALL patients with complete remission compared with controls.^   18^

## CONCLUSION

 Both the number and function of Th22 cells change in leukemic patients suggesting that these Th subsets are implicated in the pathogenesis of leukemia, and may be a conceivable biomarker to assess patients at risk or even might be a novel approach for therapeutic intervention. However, the precise roles of Th22 cells in the pathophysiology of specific types of leukemia remain unclear and further studies are required for clarifying the accurate role of Th22 and IL-22 in each type of this disorder.
